# Supplementary material for: Antimicrobial resistance gene lack in tick-borne pathogenic bacteria
Source: Sci Rep. 2023 May 20;13:8167. doi: 10.1038/s41598-023-35356-5 (PMC10199904; doi:10.1038/s41598-023-35356-5)
Supplement: Supplementary file 1 — Supplementary Information 1. [file 41598_2023_35356_MOESM1_ESM.pdf]

Supplementary information

Supplementary File 1: Tables for the metadata on each samples analysed in this study. Samples from the included genera are placed in separate sheets in the excel file. The tables contain the NCBI BioSample ID (column BioSample ID), the sample origin (column sample_origin), sample host (column sample_host), country (column country) and species (column species) metadata information available for each sample at the NCBI SRA database, the origin category (column Origin) samples were enrolled based on the available metadata and if the sample was included in the analysis with the KmerResistance tool (column kmerresistance).

Supplementary File 2: Tables for the predicted ARGs by RGI. Results for each genera are located in separate sheets in the excel file. The columns correspond to the BioSample ID (column BioSample ID), the ARGs name as in the CARD database (column Best_Hit_ARO), the identity and coverage estimated by RGI (columns Identity and Coverage), the gene family available for the ARG in the CRAD database (column AMR Gene Family), the predicted plasmid origin of the contig the ARG is located at (column Contig Plasmid Origin), the type of the phage sequence if the ARG was found within one (column Associated Bacteriophage Sequence) and the integrative mobile genetic elemnt the ARG was found to be associated with (column iMGE).

Supplementary File 3: Tables for the number of positive and negative samples for each species. Separate sheets are used for each genera included in our study. In the case of *Francisella tularensis* each supspecies was considered separately if that data was available. Positive and negative samples were counted for *F. tularensis* with and without considering FTU-1 as well. Columns correspond to the name of the species (column Species), the number of negative samples (column Number of negative samples) and the number of positive samples (Number of positive samples).

Supplementary File 4: Supplementary Figures 1-8 and their associated legends.

Supplementary File 5: Tables for the results of the AMRFinderPlus tool. Results for each genera are located in separate sheets in the excel file. The columns correspond to the BioSample ID (column BioSample ID), the gene symbol and sequence name as availabel in the NDARO database (columns Gene symbol and Sequence name) and the coverga and identity as estimated by the AMRFinderPlus tool (columns Coverage and Identity).

Supplementary File 6: Tables for the results of the KmerResistance tool when used with the CARD database. Results for each genera are located in separate sheets in the excel file. The columns correspond to the BioSample ID (column BioSample ID), the header for the ARG in the CARD protein homolog model database fasta file (column CARD database fasta header) and the template length, template coverage, template identity, query identity and query coverage as presented by the KmerResistance tool (columns Template_length, Template_Identity, Template_Coverage, Query_Identity and Query_Coverage).

Supplementary File 7: Tables for the results of the KmerResistance tool

when used with the NDARO database. Results for each genera are located in separate sheets in the excel file. The columns correspond to the BioSample ID (column BioSample ID), the header for the ARG in the NDARO database fasta file (column NDARO database fasta header) and the template length, template coverage, template identity, query identity and query coverage as presented by the KmerResistance tool (columns Template_length, Template_Identity, Template_Coverage, Query_Identity and Query_Coverage).

Supplementary File 8: Tables for the NCBI BioProject and corresponding BioSample IDs of the samples that were included in the present analysis. Samples from the included genera are placed in separate sheets in the excel file. The columns correspond to the NCBI BioProject ID (column BioProject ID) and the comma separated list of the associated NCBI BioSample IDs (column BioSample ID) that were included in our analysis.
